# *“In times of stress, it is good to be with them”*: experience of dog owners from a rural district of Sri Lanka

**DOI:** 10.1186/s12889-022-14863-6

**Published:** 2022-12-19

**Authors:** Devarajan Rathish, Jayanthe Rajapakse, Kosala Weerakoon

**Affiliations:** 1 Department of Veterinary Pathobiology, Faculty of Veterinary Medicine and Animal Science, University of Peradeniya, Peradeniya, Sri Lanka; 2 Department of Family Medicine, Faculty of Medicine and Allied Sciences, Rajarata University of Sri Lanka, Saliyapura, Sri Lanka; 3 Department of Parasitology, Faculty of Medicine and Allied Sciences, Rajarata University of Sri Lanka, Saliyapura, Sri Lanka

**Keywords:** Child’s preference, Dog companionship, Mental satisfaction, Pet dog, Stress reduction, Village dog

## Abstract

**Background:**

The dog has been man’s best companion since ancient times. And, dog ownership is associated with improved physical activity and cardiovascular health. We aim to explore the experience of dog owners on dog ownership and its effects on personal and family health in Anuradhapura, Sri Lanka. Such studies are scarce in developing countries and rural regions.

**Methods:**

A qualitative study was conducted using in-depth interviews with dog owners in the Anuradhapura district, Sri Lanka. Interviews were tape-recorded and transcribed and thematic analysis was performed.

**Results:**

The study findings were reported under the themes of experience of dog owners, pet dogs for children, village dogs as pets, the role of pet dogs in personal and family health, and participants’ advice on dog ownership. Participants highlighted companionship as a positive aspect of pet dogs. However, expenses and reduced travel were the concerns of dog ownership. A child’s preference was important in owning a pet dog. Also, participants were willing to adopt the village dogs. Further, they perceived stress reduction and mental satisfaction when interacting with their pet dogs. Moreover, participants seldom experienced major health risks from their pet dogs.

**Conclusions:**

Human–dog interaction seems to improve the participants’ mental well-being, and future research should focus on its possible consequences. Further, the village dogs were adopted as pets. Guided promotion of such activities could ease concerns related to village dogs in developing and rural regions.

## Background

Pets are considered valued family members [1] and are known to influence families throughout life [2]. Also, pet ownership is associated with human physiological, psychological, and social benefits [3]. Further, experimental studies provide evidence for human physical health benefits from human-animal interaction [4, 5]. Moreover, pet companionship and socialization improve human mental health [6]. Nevertheless, pet ownership may carry health risks for humans including injuries, zoonosis, and grief following a pet’s death [7, 8]. Since ancient times, dogs have been man’s best companions [9].

'One health’ concept identifies the human-animal bond as a way to improve mental well-being [10]. Dog ownership is associated with positive health benefits [11, 12]. Evidence suggests that dog ownership improves physical activity and cardiovascular health [13–16]. Dog owners who regularly walked their dogs had a lower chance of self-reported diabetes, hypertension, and hypercholesterolemia compared to non-dog owners [17]. Also, social support via pet ownership reduced blood pressure response to mental stress [18]. A study among children aged 5 to 17 years has shown that pet ownership reduces the chance of hypertension [19]. Also, a study among youths between 9 and 19 years with type 1 diabetes showed that children caring for pets at home are more likely to have control over their glycemic levels than children who do not care for a pet [20]. Further, pet ownership showed lower serum triglycerides [21] and low-density lipoprotein cholesterol [22]. Yet, dog owners tend to delay their medical care due to their pets [23].

Thus, it is essential to have background information on the effects of a pet dog on personal and family health to implement one health via human-animal interaction. Most of the studies on the above concept are reported from the Global North. Studies from developing countries and rural regions are scarce. Such findings are essential to evaluate and implement future strategies on human health benefits from human–dog interaction in those regions. Hence, we aim to explore the experience of dog owners on dog ownership and its effects on personal and family health in Anuradhapura, Sri Lanka.

## Methods

### Study design, setting, and population

To achieve the study objective, a descriptive phenomenological qualitative study design using in-depth interviews with dog owners was conducted. Further, the interaction between the owner and the dog was observed during the interview. The above design helps to understand the subjective experience and it is commonly used to describe the lived experience of individuals in social and health sciences-related qualitative research. However, the data collection and analysis are time-consuming and laborious. And, the results require interpretation without researcher bias [24, 25]. The study was conducted in the Anuradhapura district, a rural [26], agrarian [27] district that is the largest by surface area in Sri Lanka. The dwellers of the district during the study period were considered the study population.

### Selection criteria and sampling method

Dog owners aged ≥ 40 to ≤ 65 years, permanently residing in Anuradhapura district for ≥ 5 years, owning ≤ 3 dogs, and did not own any other pet animal for the last 1 year were recruited as participants. The above group of dog owners was selected as they are ideal informants to interview about the effects of a pet dog on personal and family health. The experience gained by the investigators during a previous project conducted in Anuradhapura, Sri Lanka, on the prevalence of pet ownership was used to recruit the first participant [28]. The next wave of participants was formed using the snowball sampling method to locate information-rich key participants. Data collection was continued until data saturation was achieved in the last three interviews. Interested participants discussed the study with the principal investigator (first author) over the phone before fixing a time for an interview. The approach was adopted to suit the present study from previously published similar literature [29].

### Study instruments

Following basic demographic data were collected: divisional secretariat (DS) division, owner’s gender, owner’s age in years, owner’s occupation (employed/unemployed/retired), responsibility for pet care (individual/shared), number of dogs owned, dog breed and dog gender. An interviewer guide (Table 1) was developed according to previously published similar literature [29] and standard guidelines [30]. The guide for the in-depth interview consisted of open-ended questions regarding the experience of dog owners on dog ownership and its effects on personal and family health. The guide was developed in Sinhala as the high majority of the target population is Sinhalese and the in-depth interviews were conducted in Sinhala. The study instruments were pilot tested on three individuals from different households.

**Table 1:** The interviewer guide for the in-depth interviews

*No*	*Item*
***Open-ended questions***	
1	What are your reasons for owning a pet dog?
2	How do you feel now about owning a pet dog?
3	What are the positive aspects of owning a pet dog?
4	What are the negative aspects of owning a pet dog?
5	What are your general thoughts on owning a pet dog?
6	What are the health benefits of your pet dog?
7	What are the health risks of your pet dog?
8	What is your prior knowledge of the effect of a pet dog on health?
9	Do you have any other special messages?
***Observations on the interaction between the owner and the dog during the interview***	
1	Interaction and gestures between the owner and dog
2	Physical space between the owner and dog
3	Owner’s expression when he/she refers to the pet dog
4	Owner’s expression when the pet dog is physically present
5	Dog’s expression when the owner refers to the pet dog
6	Dog’s expression when the owner is physically present
7	Level of attention received by the dog from the owner
8	Level of attention received by the owner from the dog

### Data collection

Data collection was conducted from November 2021 to January 2022. The interviews were conducted at a convenient time for the participant at his / her private residence, allowing the pet dog to be present during the interview. Information about the study was explained by the principal investigator (first author) and informed written consent was obtained before the commencement of each interview. The interview was conducted in the Sinhala language for 20 to 30 min duration and was audiotaped with permission. An interviewer (principal investigator) and a trained note keeper were present during the interview. At the end of the interview, respondent validation was achieved by discussing and finalizing the collected information. Training, in-depth interviews and data documentation were done according to standard guidelines [30].

### Data description and analysis

Audiotapes of each interview were transcribed and typed into a Microsoft Excel sheet by the principal investigator (first author). Backup copies of audiotapes were securely stored in a separate folder. Field notes and notes on observations for the interaction between the owner and the dog during the interview were typed up and added to the same sheet. Backup copies of transcripts were securely stored in a separate folder. Demographic data were presented as percentages and thematic analysis was performed manually by pile sorting for the transcribed recordings. Each transcript was coded by two investigators independently then discussed similarities and differences in coding to form the code list. We aimed to utilize the diverse perspectives of the two independent coders and discover new codes. The final codes were categorised into themes. Participants were contacted over the phone to discuss themes derived from the data. Security and confidentiality of data were maintained at all times. All relevant files were stored in a password-protected computer. Field notes and notes on participant observations were protected under lock and key. Data transcription of recordings, data organization, and data storage were done according to standard guidelines [30].

## Results

### Demographic data

**Fig. 1:**
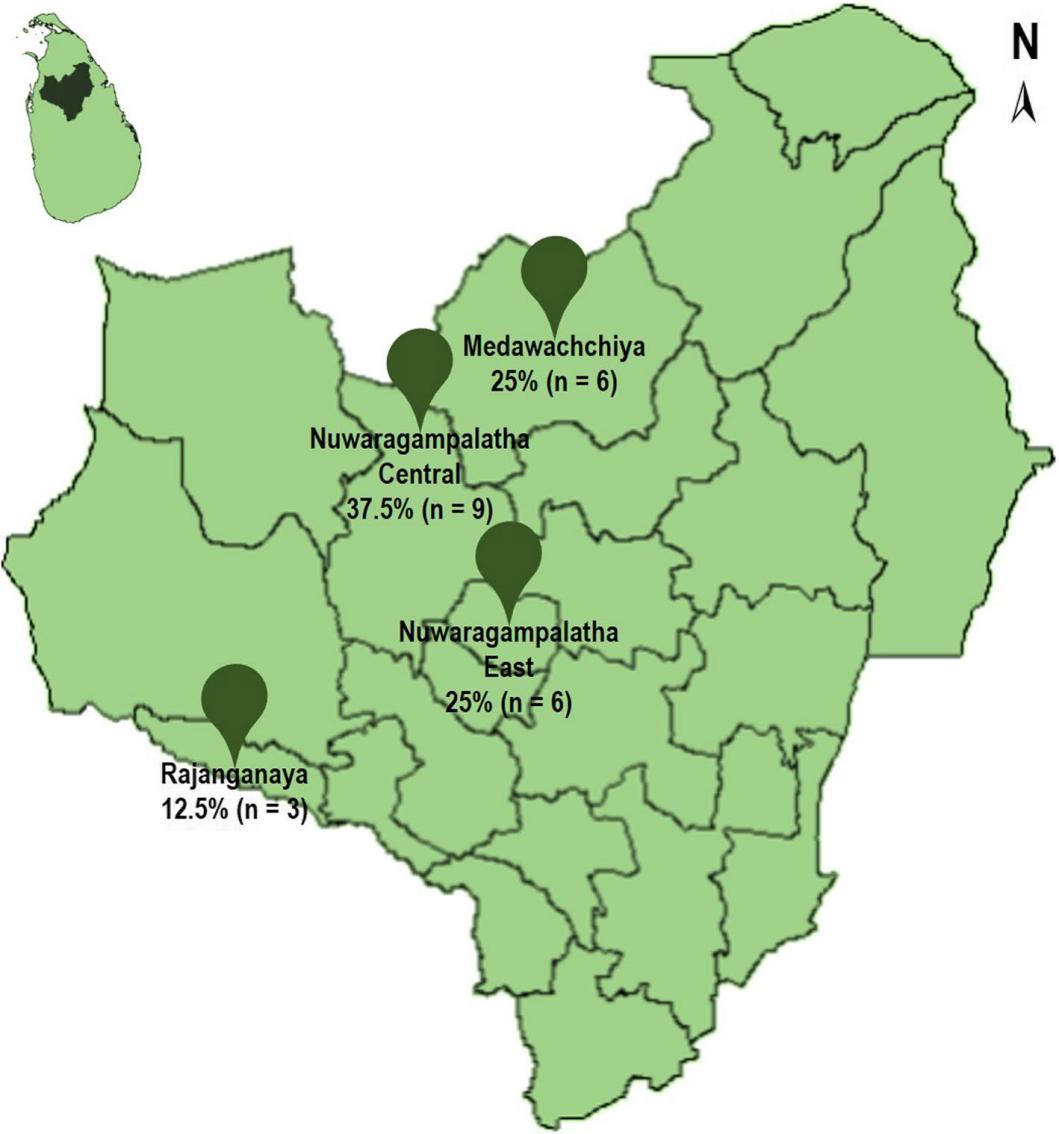
The map showing the divisional secretariat (DS) divisions of Anuradhapura district of Sri Lanka where the participants resided and the number of participants selected with percentages

**Fig. 2:**
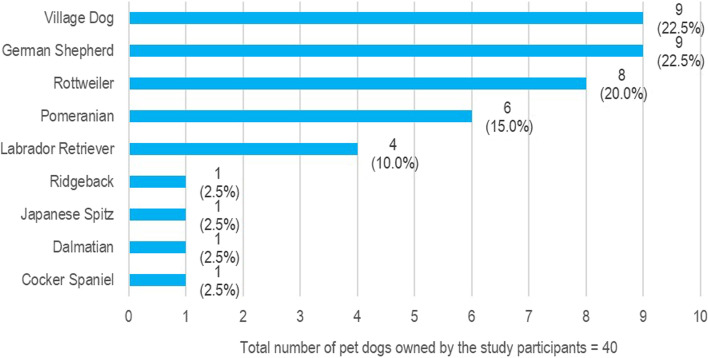
Pet dog breeds owned by the participants

A total of 26 pet dog owners were approached and data from 24 participants were collected (92.3%). Two owners refused participation as they were busy with their day-to-day activities. The mean age of the participants was 51.6 (SD 6.7) years. Most were females (54.2%—13/24), unemployed or retired (62.5%—15/24), from Nuwaragampalatha Central DS division (37.5%—9/24) (Fig. 1), sharing responsibility for pet care (87.5%—21/24), and having only one pet dog (50%—12/24). The participants owned a total of 40 pet dogs out of which 57.5% (23/40) were female dogs and 42.5% (17/40) were male dogs. The most common pet dog breeds were the village dog (22.5%—9/40), and the German Shepherd (22.5%—9/40) (Fig. 2). Demographic data are summarized in Table 2. Five themes emerged from the experience of dog owners on dog ownership and its effects on personal and family health. The final codes and the categorized themes are shown in Table 3.

**Table 2:** Summary of participants’ demographic details

Participant code	Owner's gender	Owner's age	Owner's occupation	Responsibility for pet care	No of dogs	Dog breed	Dog gender
A	F	44	Unemployed	Individual	1	Cocker Spaniel	M
B	F	55	Unemployed	Shared	2	A—Labrador Retriever, B—Pomeranian	M, F
C	F	40	Unemployed	Individual	2	A—Rottweiler, B—Pomeranian	M, M
D	M	61	Retired	Individual	3	Village dogs	F, F, F
E	M	47	Employed	Shared	1	Village dog	M
F	M	52	Employed	Shared	1	German Shepherd	F
G	M	58	Employed	Shared	1	German Shepherd	F
H	F	55	Unemployed	Shared	2	A—German Shepherd, B—Pomeranian	M, M
I	M	53	Retired	Shared	1	German Shepherd	F
J	F	50	Retired	Shared	1	Labrador Retriever	F
K	M	60	Employed	Shared	1	German Shepherd	M
L	F	50	Unemployed	Shared	1	Village dog	M
M	F	43	Unemployed	Shared	2	Village dogs	F, F
N	M	60	Retired	Shared	1	Dalmatian	M
O	F	60	Retired	Shared	1	Village dog	F
P	M	49	Retired	Shared	2	German Shepherds	M, F
Q	F	54	Unemployed	Shared	2	A—Ridgeback, B—Village dog	M, F
R	F	42	Employed	Shared	2	A—Japanese Spitz, B—Pomeranian	M, M
S	M	51	Employed	Shared	3	Rottweilers	M, F, F
T	F	49	Unemployed	Shared	1	Rottweiler	F
U	F	40	Employed	Shared	1	Rottweiler	M
V	M	55	Employed	Shared	3	A—Rottweiler, B—German Shepherd, C—Pomeranian	F, F, F
W	M	62	Employed	Shared	2	Labrador retrievers	F, M
X	F	49	Retired	Shared	3	A—German Shepherd, B—Rottweiler, C—Pomeranian	F, F, F

*M* Male, *F* Female

**Table 3:** Themes and codes

*No*	*Themes*	*Codes*
1	Experience of dog owners	Companion Sense of security Expenses Reduced travel Difficulty in keeping the residence clean Reduced transactions with neighbours
2	Pet dogs for children	Owned due to child’s liking Child’s physical well-being Child’s mental well-being Reduced addiction to the internet
3	Village dogs as pets	Adopting village dogs Difficulties in retaining them at home
4	The role of pet dogs in personal and family health	Mental well-being Mental distress on a pet’s illness or death Physical well-being Spread of infections Allergies Less time to spend on health
5	Participants’ advice on dog ownership	A learning experience Easy and enjoyable occupation A personal choice A responsibility Do not cage a pet dog A need for a separate crematorium for pet dogs

### Observations on the interaction between the owner and the dog during the interview

**Fig. 3:**
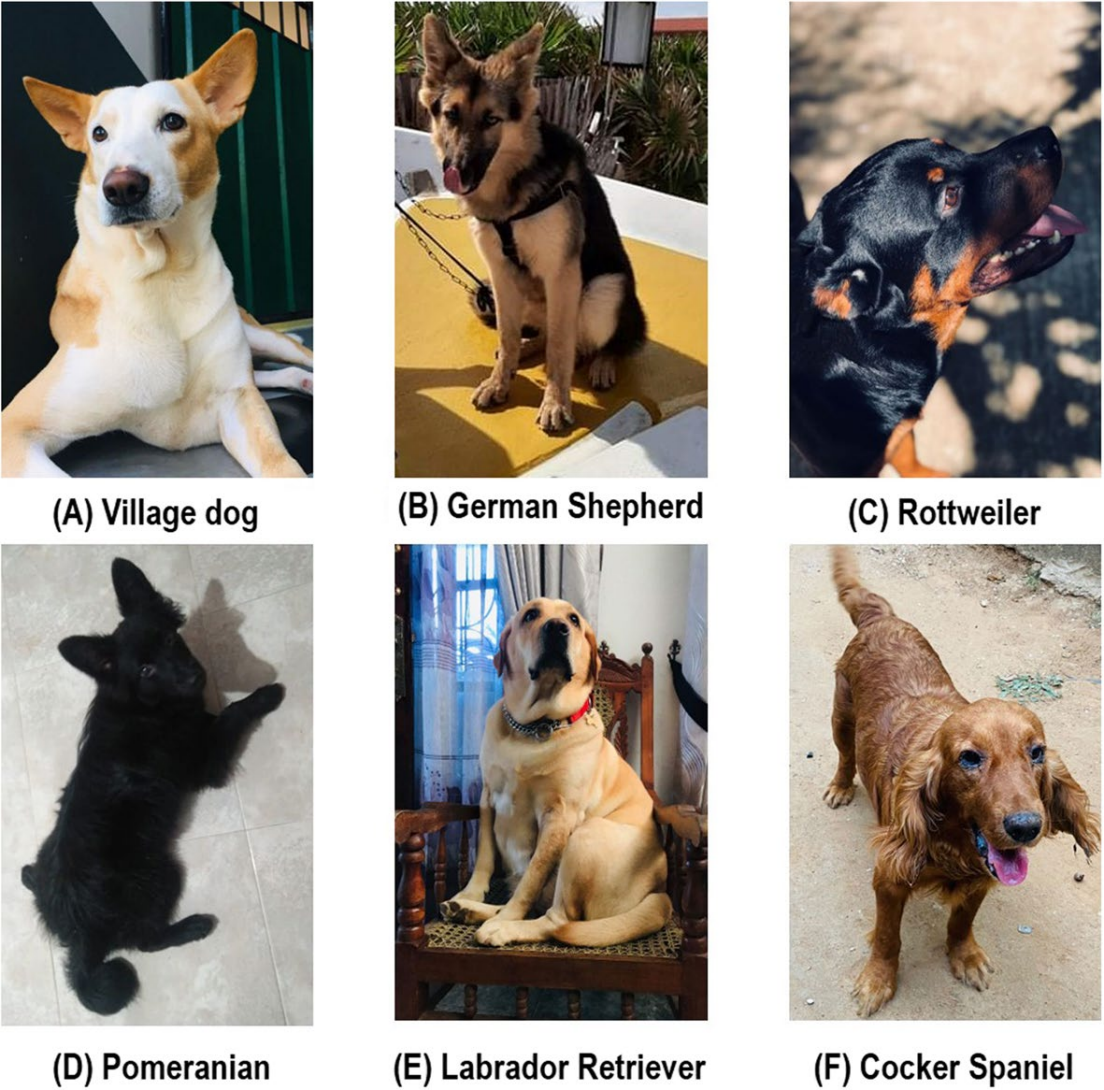
Images of the pet dogs provided by a few participants

The participants had verbal and non-verbal communication and close interaction with their pet dogs. Participant U called her dog by name and gestured to hold her hand. The dog obliged and held her arm. Both were in a playful mood during the interview. Also, the participants were proud when they referred to their pet dogs. Participant I had a father-like attitude towards his pet dog. He was proud when he referred to his dog and was stroking the pet dog with love and care when the dog was physically present. The pet dogs had a wide range of expressions like attentive, docile obedient and playful when the owner referred to the pet dog. Also, pet dogs had a wide range of expressions like adorable, agile, exuberant, playful, protective and tail-wagging when the owner was physically present. Thus, the participants had an excellent level of attention towards their pet dogs and received an excellent level of attention from their pet dogs. Images of the pet dogs provided by a few participants are shown in Fig. 3.

### Experience of dog owners

Participants highlighted petting and companionship as positive aspects of pet dogs. Participant J informs *“she is a pet and a family member”*. Also, participants felt happy and loved owning a pet dog. Participant B says *“I love it. I can take care of even one or two more. I like to bathe and powder them. It is a different world to be with them. I love what they do. When we call by name, they recognize, get up and come to us”.* Participant L notes *“he is obedient like a child and listens to what we say”* and participant C emphasized that *“I could wait without seeing my children, but I cannot wait without seeing them (pet dogs)”*. Further, participant R elaborated *“They benefit more than my children. When travelling by car they are in the front seat. They sleep in the best place. While we are eating have to share with them”*.

Feeling secure is an important aspect highlighted by participants. Participant M says *“(They were) brought for security and to pet. I like to look after them”*. And, participant A said that *“due to his (pet dog’s) aggression, usually people do not come without calling. Therefore, I think he is good to be there”*. And, participant M added that pet dogs are *“good for security. Even if a snake enters, they bark”*. Moreover, participant S showed his feelings as *“really happy. They (pet dogs) show more gratitude than a human being”*. Also, participant I mentioned, *“when we place a baby on the cradle, she (pet dog) sits beside and does not let anyone go near. When the baby cries, she comes to tell us”*. And he continues to praise his dog *“If we go to the lake to bathe, she will not allow us to go deep into the water. She hangs by our hand until we come up”*. Further, participant R narrates *“if we send the driver home to collect something, he (pet dog) will not let the driver in when we are not there. But, *via* the speaker on the phone, if he was told to allow the driver in, he would step aside and do so”*. However, participant C says *“as they were brought up as pets, nothing can be expected on security. Recently there was a house burglary while these two were inside the house. Someone whom we knew had come during the day. They do not bark at known individuals”*.

Participants have highlighted expense and reduced travel as negative aspects of owning a pet dog. Participant X expresses her concerns *“We can't leave them and go anywhere. They are too big and we can’t take them with us. Travelling has reduced. Have to spend around 40,000 rupees a month for food and health”*. However, she explains how she could meet her additional expenses for the pet dogs *“once we have the puppies, we will be able to cover our expenses”*. Also, some participants struggled to keep their residences clean. Participant T confirms it by saying *“the house is dirty. And, the dust enters in. Fur (of the pet dog) falls a lot. Have to mop the house frequently”*. Further, there were negative feelings expressed by participant J who lamented *“lots of losses. I do not know if it is due to my lack of experience in petting a dog. She eats our slippers and clothes. You have to spend a lot of time with her. I feel a lot. We need to feed her before we eat”*. Moreover, participant A reveals her trouble *“transaction with neighbours have also declined”*.

Companionship and security from the pet dogs were strongly echoed as positive experiences by the participants. While, expenses, reduced travel, difficulty in keeping the residence clean, and reduced transactions with neighbours were highlighted as negative experiences of dog owners.

### Pet dogs for children

A child’s preference was an important factor in bringing home a pet dog. Participant C said *“my little one looks at the phone and searches for information about dogs”* and participant J explained that *“when my son got married, my daughter was alone. When my daughter’s ordinary-level examinations were over, the dog was brought home for her company. She (the pet dog) was brought for my daughter’s physical and mental health as she does not play with other children these days (due to the COVID pandemic)”*. And, participant P pointed out an important effect of a pet dog on a child’s mental health as follows *“with natural things like this (pet dogs) we can reduce the addiction to the internet among children”*. Hence, dog ownership due to the child’s liking was highlighted by participants. Also, physical well-being, mental well-being, and reduced addiction to the internet were the benefits noted by owners for their children from their pet dogs.

### Village dogs as pets

Participant O had an interesting statement on how she found her village dog *“we found her by chance. She had an accident in front of our house. She was brought in, treated, and is now being cared for”*. Participant D was proud in saying *“in the past when I had a lot of (village) pet dogs, they come in front of my car to the junction like an equestrian parade. They do not allow people to come, and they do not allow dogs to come. They bark at them. Once I leave, they come back home”.* Participant O enlightens us by saying *“most breed dogs are owned for a show. Adopting is better. Normal (village) dogs fall ill on the road. It is better to take care of them (village dogs) than to buy dogs for money. It's good for them (village dogs) too”*. And, participant K firmly advises us *“it is not good to let a pet stray”*. Further, participant S confirms the above statement by saying *“if possible, give a meal to a street dog at least once. You will observe it come (to you) wagging its tail”*. However, participant G says *“Previously we had village dogs. Their presence is not useful. They go here and there”*. Thus, the study participants emphasised adopting village dogs but some experienced difficulties in retaining them at home.

### The role of pet dogs in personal and family health

Stress reduction and mental satisfaction were felt by participants. Participant C says *“Even when I have a problem in my mind when I feel stressed, it feels like the problem and stress in my head is lessening while I am petting and talking to them”*. And, participant D adds *“It is for mental health. In times of stress, it is good to be with them”*. Participant I confirms the above by saying *“there is one hundred per cent mental satisfaction”*. Also, participant N points out that it is *“good for son's mental health”*. Participants mentioned that they experience mental distress when their pet dog is ill or dead. *“We love him so much. Therefore, we would feel sad if he falls sick”* states participant U. Participant D talks about the death of his dog *“the only thing that is a little problematic is the grief when the dogs die. They are with us for about ten years. We bury them in the rear garden. We organize an alms-giving too”*.

Participants mentioned that they engaged in physical activities along with their pet dogs. It is evident in participant W’s statement *“there is peace of mind when they are there. We go to the ground in the morning, to walk and play. If they see somebody, they want to play”*. However, participants were worried about upper respiratory tract-related issues and skin allergies. Participant C says *“both daughters have got allergies. The two dogs climb into the bed. A skin allergy. It does not go away even after treatment for a long-time. When channelled (a doctor) we were told to keep the pet dogs away. Also, they (daughters) develop a frequent cold”*. And, participants highlighted the issue of the spread of fleas. Participant F tells us that the *“spread of fleas is too high in our area. She (pet dog) gets it from the other dogs”*. Participant J had a different perception about the health risks of her dog and she says *“after Eli came home, my health began to decline. I could not do meditation or yoga. I have less time for my work. I gained weight. Thyroxine drug was started. I needed medication for cholesterol too”*. However, participant B declares that *“I have had diabetes for thirteen years. The control did not change just because a pet dog was brought”*. Participant T had something interesting to say *“as the dog approaches, my husband gets a bad smell. He doesn't like it. Sometimes his pressure might have increased”*. However, participant E says *“We have not had any health issues from our pet dog. He does not come into the house. Even when we come in after playing with him, he stays out”.* And, participant H confirms it by saying *“there is no such thing”.* However, participant D tells us that *“we did not think much about it. We did not think of the health benefits for us. Thought highly of their (pet dogs) health”*.

Participants had known about the risk of rabies. Participant K said, *“if there was a rabid dog, it can bite us and harm nearby families too”*. And, participants knew that the fur of the pet dogs leads to wheezing. Participant V stated, *“I have heard that due to fur children get a wheeze. I have not heard the effect on mental health”*. Further, participants had heard of the spread of infection through pet dogs. Participant T states *“I have heard that there can be a spread of infection”*. Moreover, the following participants had unique knowledge to share with us. Participant J told us *“I knew it would help my daughter's mental and physical health. I heard from others”*. And, participant O says *“I knew *via* the internet it (having pet dogs) is good for mental stress”*. While participant S mentioned that *“I have heard *via* the internet that having pet dogs could lead to arthritis”*. However, some participants had no prior knowledge of the effect of a pet dog on health. Participant Q confirms it by saying *“we had no (knowledge) as such”*. The participants continue to be compassionate towards their pet dogs as the benefits they perceive overweighs the risks.

**Fig. 4:**
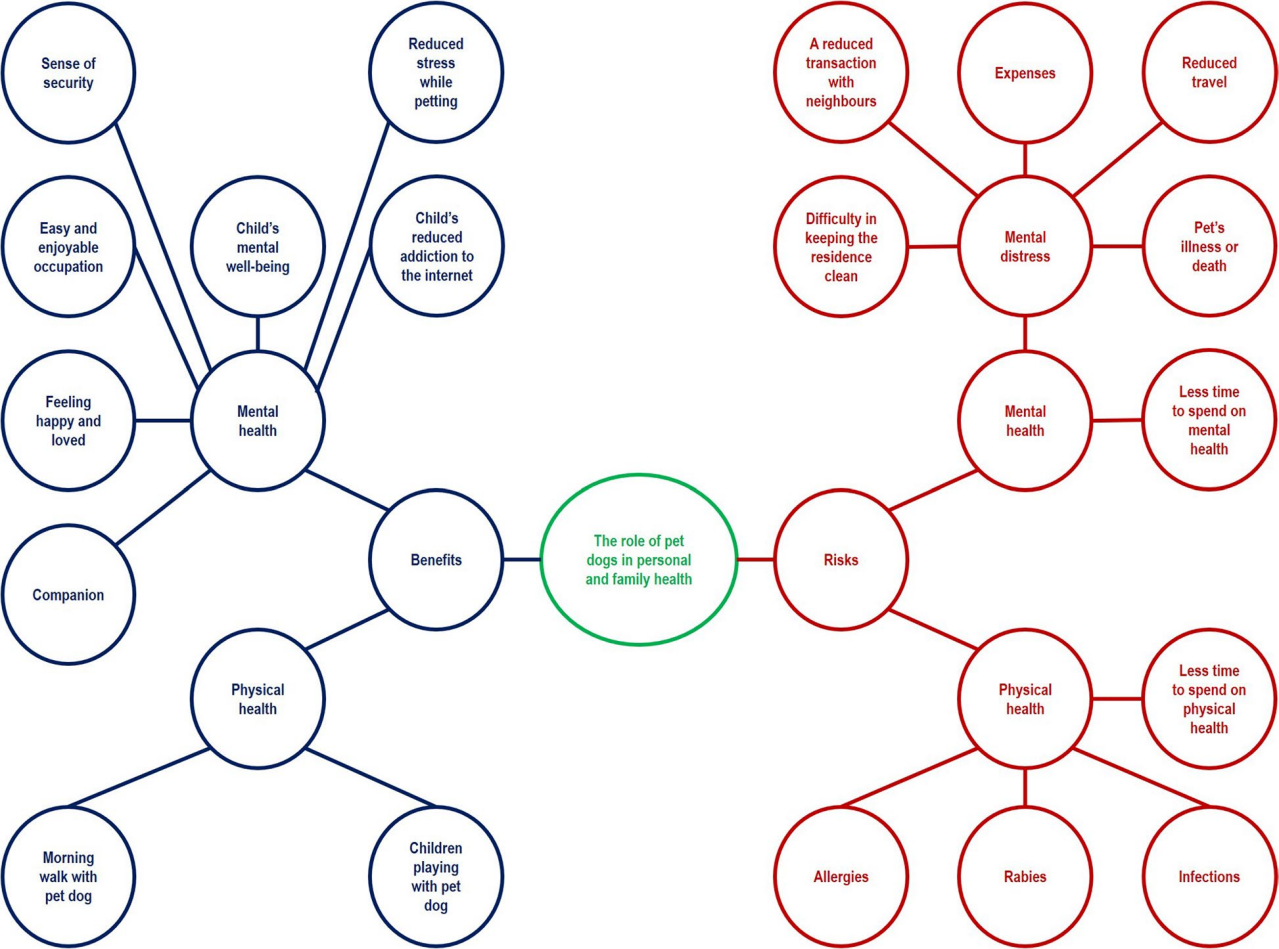
The mind map on the role of pet dogs in personal and family health

Physical and mental well-being were health benefits emphasized by dog owners. However, mental distress by a pet’s illness or death, the spread of infections, allergies and less time to spend on health were pointed out as issues by a few participants. The mind map developed on the role of pet dogs in personal and family health is shown in Fig. 4.

### Participants’ advice on dog ownership

“This is a different experience for those of us who have a monotonous life”, says participant E. And, participant F adds “you can learn a lot while being with them”. However, “if we want a quiet environment and want to read books, we do not need a pet. An unnecessary bond. A pet is a hindrance if you feel the need to reduce ties. Having a pet is good if you want companionship” recommends participant J. And, participant G is confident in saying “it (having a pet dog) is not difficult. It is easy”. Participants emphasized that having a pet dog is a personal choice and cannot be forced. In general, participants thought that the owners should bear the expenses and should take proper care of their pet dogs. And, participant F accentuates that “something bigger than you think. It is quite a responsibility. It is like taking care of a little child. It is harder than that. A child can be kept with someone while you are out. But they (pet dogs) cannot be”. Participant R tells us to “bring up a pet dog only if possible. There is no point in bringing up animals with the idea to attack and harass. Have to feed them. Give your love (to your pet dog) like for children. There is no point in bringing up animals trapping their world by tying them in chains. They have the same needs as we do. They have feelings. They show love. It is felt by the ones who closely associate with them. There is no point in feeding them twice a day, tying them by a chain, caging them, and beating them when they urinate or poop. Bring up only if possible. In terms of cost, the amount is higher. For food and medicine”. Participant W concludes “when pets are lost, they need a separate crematorium of their own. They have been living with us in the same family for many years. A separate place is needed for the last rights without dumping them in a garbage pit”.

Dog ownership was described as a learning experience, an easy and enjoyable occupation, a personal choice, and a responsibility by the participants. They advise not to cage a pet dog and felt the need for a separate crematorium for pet dogs**.**

## Discussion

Our study explores the experience of dog owners on dog ownership and its effects on personal and family health in Anuradhapura, Sri Lanka. The above is a less studied area in developing countries and rural regions. Owners were observed to have an excellent level of attention towards their pet dogs and received an excellent level of attention from their pet dogs. The study found that participants experienced stress reduction and mental satisfaction from interacting with their pet dogs. And, they seldom experienced major health risks from their pet dogs. Similarly, a Japanese study revealed that dog ownership had a positive effect on mental well-being [31]. And, pet ownership was associated with greater physical activity and better mental health during the COVID-19 pandemic in Singapore [32]. Also, a meta-analysis revealed a significant difference in self-reported stress after pet therapy [33]. However, a systematic review found variable results such as positive, mixed, negative, and no impact of pet ownership on mental health [34]. Thus, an objective assessment of the association between dog ownership and mental well-being is warranted.

Children at home tend to play a major role in pet dog keeping. A child’s preference was an important factor in bringing home a pet dog. Also, participants felt that having a pet dog would contribute to a child’s physical and mental health. Further, the owners assume the child’s addiction to the internet can be reduced by having a pet dog at home. An American study showed that higher child-to-pet dog attachment is associated with increased child physical activity [35]. And, a prior systematic review had evidence for an association of pet ownership with emotional health, educational and cognitive benefits [36]. Also, a Japanese study suggests that owning pets might help children to control their emotions and reduce poor emotional expression [37]. Although evidence on the role of pet dog ownership in internet addiction is scarce, there has been an instance where the Korean government has offered pets for internet addiction [38].

Village dogs were the most common pet dogs along with German Shepherds. The village dogs seem to be an inexpensive choice in the rural setting. And, participants were willing to adopt the village dogs who were found on the roads or who needed help after an accident. However, a few participants thought village dogs are not useful as they could leave the household. Studies on village dogs as pets were scarce. A study from rural western Uganda indicates that infectious disease is important to the health and survival of village dogs, and, ownership and interactions with wildlife contribute to morbidity and mortality [39]. Further, free-roaming domestic dogs in rural communities of Tanzania were found to exist in the context of their human owners and surrounding wildlife [40]. Moreover, village dogs in the Global South are a concern for people, tourists and organizations. Overpopulation, welfare, zoonoses, and predation of wildlife are a few concerns [41]. Adopting village dogs as pointed out by our study participants might help ease such concerns. However, experts are divided in their opinion on adopting village dogs as pets. Some argue that the village dogs are self-sufficient, social animals who do not need human interference to save them. While others say that village dogs can successfully be adopted as they are not wild [42]. Also, online step-by-step guides are available for adoption which could help dog lovers in adopting a village dog [43, 44]. However, the lack of reliable indicators of quality in online information would be a barrier to dog owners’ access to reliable information [45] in addition to the lack of internet access in developing and rural regions.

Dog ownership was not without concerns for the owners such as expenses, reduced travel, and struggle to keep the residence clean. Some have experienced grief due to the loss of a pet. While others were worried about upper respiratory tract-related issues, skin allergies and the spread of fleas. Prior literature outlines similar drawbacks in pet ownership such as grief due to pet loss, fear of outliving the pet, pet care as a chore, risk of falls, cost, and the fear of leaving a pet due to relocation [46–48]. Responsible pet ownership should be promoted where careful selection of pet(s) suited to your home, lifestyle and family members is advised [49]*.* Also, local norms have to be considered before the promotion of pet ownership since factors such as households with more than one adult female and Buddhists were significantly associated with dog ownership in Anuradhapura, Sri Lanka [28]. Further, a review revealed dog’s physical appearance, dog’s behaviour and health, social influences, the owner’s demographic and socioeconomic factors, and the owner’s previous ownership experience are factors associated with acquiring a pet dog [50]. In addition, checking for friends/family or daycare facilities that can care for pets in their absence, employing hygiene practices and educating children about safe interaction are a few practices to mitigate negative aspects of dog ownership [51]. Moreover, the globalization-related flow of goods, services, technology, and information could impact decision-making on pet dog ownership. Integrated research on social, economic, and environmental factors is proposed to address issues related to exotic pets’ implications on the conservation of native biodiversity and the emergence of invasive species [52].

Experience of dog ownership and their influence on health could be different by geography, religion and ethnicity. The present study included only Sinhalese from Anuradhapura due to travel, time and resource constraints. Evidence could have been strengthened by videotaped observation of owner-dog interaction despite the challenges of consent and data analysis. Further, the present study selected only the later middle age group of ≥ 40 to ≤ 65 years as participants. The experience of younger and older dog owners would differ from the present findings. Pets often form an important source of social support in later life; this is an important area for future research. Nevertheless, the findings are unique because it was conducted in a rural district of a developing country where similar studies were scarce. The perceived stress reduction and mental satisfaction among dog owners will be useful in planning out research and intervention related to one health. Also, the willingness to adopt village dogs should be promoted with careful guidance. Future research should focus on ways to implement responsible pet ownership among residents in rural regions with pet dogs. Also, the sterilization service from the Public Health Veterinary Services, Ministry of Healthcare and Indigenous Medical Services, Sri Lanka could be utilized for feasible spaying or neutering of the adopted village dog [53].

## Conclusion

Participants perceived stress reduction and mental satisfaction when interacting with their pet dogs. Also, they seldom experienced major health risks from their pet dogs. Human–dog interaction improved the participants’ mental well-being, and future research should focus on its possible consequences. A child’s preference was an important factor in bringing home a pet dog. Further, the adoption of village dogs as pets was prominent which can be promoted to ease concerns related to village dogs in developing and rural regions. However, the implementation of responsible pet ownership is essential to address the concerns of dog owners such as expense and reduced travel.

## Data Availability

All data generated or analysed during this study are included in this published article.

## Abbreviations

DS: Divisional Secretariat
F: Female
M: Male
